# New Treatment of Carbuncle

**Published:** 1897-02

**Authors:** T. C. Osborn

**Affiliations:** Cleburne, Texas


*Editors Texas Medical Journal:*

### New Treatment of Carbuncle—Reply to Dr. Morris.

CLEBURNE, TEXAS, January 14, 1897.

*Editors Texas Medical Journal:*

The January issue of the JOURNAL afforded me an agreeable surprise in the criticism of Dr. Morris on my treatment of carbuncle.

It is especially gratifying to me to see in his paper a genial corroboration of my estimate of the therapeutical value of the "Kumpe tonic," and of the hyposulphate of soda; only that in my use of the tonic I do not limit it to its anti-malarial virtues, but prescribe it wherever I need in practice its invigorating influence, whatever the nature of the disease may be. In my estimation there is not known a more powerful medicine of its class in the whole therapeutical range, and the only objection to it is its intense bitterness to the taste. Dr. Kumpe urged its adoption as an anti-malarial agent, and unlike my waywardness, Dr. Morris has continued to restrict its usefulness to that province alone. There is no other difference on this point between us.

He objects to my etiology of the anthrax, saying my treatment of it, even down to the cinchona poultice, clearly proved that my assertion of belief that it was caused by "protracted indigestion," was a pathological paradox, and as unexplained, he has good reason for his assertion. My explanation is this way: It will hardly be denied by any one that there is barely one out of a thousand cases of carbuncle which has not a malarial background; and as to "protracted indigestion," there is not a more potent agent in its causation than malarial infection. If this predicate is well founded, my treatment of carbuncle is based upon the most rational management, and ought to supercede the old surgical interference, and remove its insertion at once from the surgical repertory. I do lay claim to priority in that respect. The only real difference in our theories of carbuncle is, my reading and experience force me to believe in its non-recurrence; while, on the other hand, Dr. Morris has had in his own person as many as three separate attacks of the painful affliction. It is very pleasurable to me to be corrected in my theories as kind Dr. Morris has done in this instance, and feel amply repaid for the mental labor undergone in hunting through libraries, and in patient inquiries on every hand, for instances of recurrence, and for the infectiousness of anthrax, under the conviction that the disease was of a germicidal character; and finally to have my theory corrected by such unequivocal proof. I am mainly sorry that I had not made Dr. Morris' acquaintance earlier in my career. He deserves and has my sincere thanks.

The strangest thing now about the affliction is, in my estimation, why the sites for its development are most frequently found on the posterior parts of the body? Some allowance is made for its appearance on the neck in males, as that position is often chafed by starched collars. And it may be that women most often have the trouble on the dorsal region because of the stricture on that region by tight dressing. But as the disease has been seen on many other parts of the body, such an inference is hardly tenable. I wish I could explain it, but I cannot.

The tonic treatment of carbuncle is by no means new, but in every instance where it is prescribed by writers, it is advised only in the last stage of its existence, where the system has been exhausted by the malignancy of the disease. It is also possible for other tonics besides Kumpe's to be equally serviceable in arresting anthrax. Of this I can offer no opinion, since I have used in that way only Kumpe by preference.

After all, I make less doubt now than ever, that the *exciting* agency of "protracted indigestion" is the true cause of anthrax; the predisponent being malarial infection; and I feel quite sure it will require very positive proof, like that given by Dr. Morris on its recurrence, to convince me of my error.

Yours very cordially,

T. C. OSBORN, M. D.

